# The Strategy and Early Clinical Outcome of Percutaneous Full-Endoscopic Interlaminar or Extraforaminal Approach for Treatment of Lumbar Disc Herniation

**DOI:** 10.1155/2016/4702946

**Published:** 2016-08-25

**Authors:** Weijun Kong, Wenbo Liao, Jun Ao, Guangru Cao, Jianpu Qin, Yuqiang Cai

**Affiliations:** Department of Spine Surgery, The First Affiliated Hospital of Zunyi Medical College, Zunyi 563000, China

## Abstract

Objective is to analyze the surgical strategy, safety, and clinical results of percutaneous full-endoscopic discectomy through interlaminar or extraforaminal puncture technique for LDH. Preoperative CT and MRI were analyzed, which were based on the main location of the herniated disc and its relationship with compressed nerve root. Sixty-two patients satisfied the inclusion criteria during the period from August 2012 to March 2014. We use percutaneous full-endoscopic discectomy through different puncture technique to remove the protrusive NP for LDH. Sixty patients completed the full-endoscopic operation successfully. Their removed disc tissue volume ranged from 1.5 mL to 3.8 mL each time. Postoperative ODI and VAS of low back and sciatica pain were significantly decreased in each time point compared to preoperative ones. No nerve root injury, infection, and other complications occurred. The other two patients were shifted to open surgery. No secondary surgery was required and 91.6% of excellent-to-good ratio was achieved on the basis of Macnab criteria at postoperative 12 months. Acquired benefits are fewer complications, rapid recovery, complete NP removal, effective nerve root decompression, and satisfactory cosmetic effect as well. This is a safe, effective, and rational minimally invasive spine-surgical technology with excellent clinical outcome.

## 1. Introduction

The spinal endoscopic technique is designed to protect spinal stiffness and dynamic structure as well as to reduce and avoid traditional open surgery-related complications [1, 2]. Endoscopic surgery has some advantages, including clear visualization, less damage to the paraspinal muscle and other normal tissues, good cosmetic effect, and reduced patient morbidity with early return to work [3, 4]. Posterolateral working channel of transforaminal endoscopic discectomy is a popular endoscopic technique [5, 6]. But it has several disadvantages, including that it can not effectively deal with lumbar disc herniation located mainly in spinal canal, complicated puncture techniques, overexposure to X-rays, and insufficient treatment of L5S1 disc protrusion [3, 6]. Another method, introduced by Ruetten et al., uses interlaminar approach into the canal; this procedure preserves the classical posterior pathway and is easy to perform, thus avoiding the insufficient treatment of L5S1 by posterolateral transforaminal approach [7]. With the development of various pathway techniques, the full-endoscopic spinal technique has become an important treatment method in intervertebral lumbar disc herniation (LDH) [3, 6–8]. Proper surgical indications and good working channel position are important for successful percutaneous endoscopic lumbar discectomy [9]. Due to limited microscopic operation space, it is necessary to obtain an accurately planted operation channel, which should approach the protrusive disc as much as possible to complete the operation. As a result, a fully exposed outstanding intervertebral disc can be viewed in limited space. We classify the primary position of the protrusive disc with compressed nerve root into the axillary type, shoulder type, and extreme lateral type. Various percutaneous targeted puncture techniques for excision of protrusive NP tissue are successful in directly decompressing the nerve root. From August 2012 to March 2014, we performed the percutaneous full-endoscopic discectomy through interlaminar or extraforaminal puncture technique to treat 62 patients with single segmental intervertebral lumbar disc herniation (LDH). The effectiveness was achieved within a clinical follow-up of more than 12 months.

## 2. Patients and Methods

### 2.1. General Data

**Figure 1:**
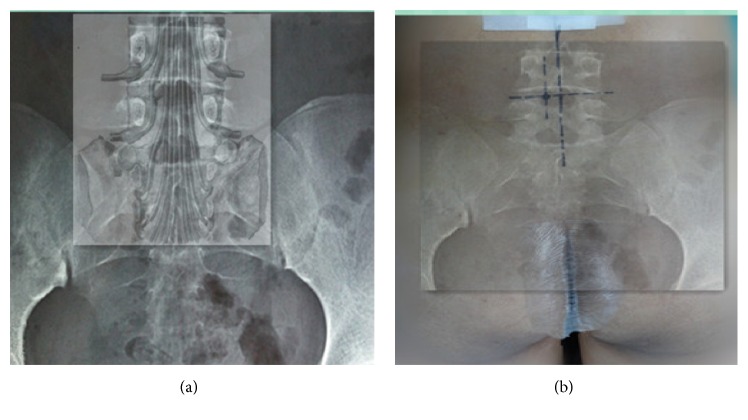
(a) Theoretical positions between the protrusive disc and the nerve root. (b) Actual puncture points on the body surface.

**Figure 2:**
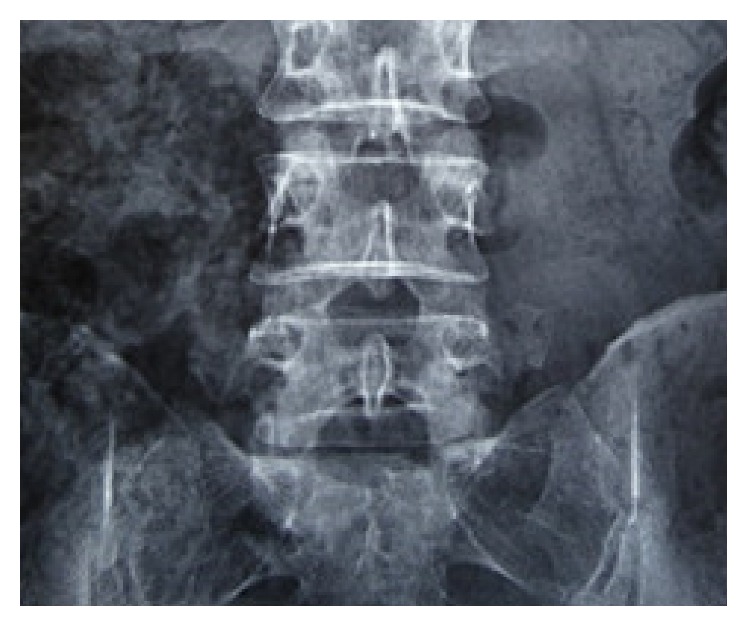
The diameter of the interlaminar window was measured as the sagittal distance between the superior edge of lower laminar bone and inferior edge of upper laminar bone and the horizontal distance between the medial of inferior facet and lateral edge of spinous process.

Among the 62 patients, 38 were males and 24 were females. The average age was 51.6 years (18–73 years). Patients had symptoms for 1 to 18 months (3.6 months on average) and were subjected to conservative treatments for at least one month. Patients with no obvious symptom alleviation and reoccurrence were selected for the operation with our protocols. The location of the protrusive disc was L3/4 (1 case), L4/5 (33 cases in total, 19 cases on the left side, and 14 cases on the right-side), and L5S1 (28 cases in total, 16 cases on the left, and 12 cases on the right). All cases were subjected to examination of the lumbar positive and lateral X-ray films, CT, and MRI. CT and MRI were requested to distinguish the position of protrusive disc and its relationship with compressing nerve root (Figure 1(a)). The results were divided into protrusive disc of the L4 nerve root shoulder type (1 case), L5 nerve root shoulder type (8 cases), L5 nerve root axillary type (22 cases), S1 nerve root shoulder type (8 cases), axillary type (20 cases), and extreme lateral type (4 cases: 3 cases of L4/5 and 1 case of L5SI). Inclusion criteria were as follows: (1) different degrees of lumbago and unilateral obvious sciatica; (2) no signs of improvement or aggravation through conservative treatment for more than 4 weeks; (3) CT and MRI revealing a single segment of lumbar intervertebral disc herniation that was not associated with bony stenosis in ipsilateral recess; and (4) lumbar anteroposterior radiographs showing that the diameter of the interlaminar window was more than 8 mm [4, 7] (see Figure 2). All patients signed the minimally invasive surgical treatment approval consent established by our hospital ethics committee after full explanation. Exclusion criteria were as follows: (1) central lumbar disc herniation; (2) local skin condition being poor and laboratory examination showing signs of infection; (3) imaging studies suggesting infection, tumor, lumbar spinal stenosis, lumbar spine deformity, and lumbar spondylolisthesis or instability [4, 8, 10]; (4) surgical history with corresponding segmental sites; and (5) abnormal blood coagulation function.

### 2.2. Surgical Instruments

The spinal endoscope system (SPINENDOS Co., Germany) comprised 4.3 mm working channel, 7 mm outer sheath diameter, and 30-degree angled scope with continuous water irrigation system.

We also used low-temperature radiofrequency ablation system (ArthroCare Co., USA).

### 2.3. Surgical Technique

**Figure 3:**
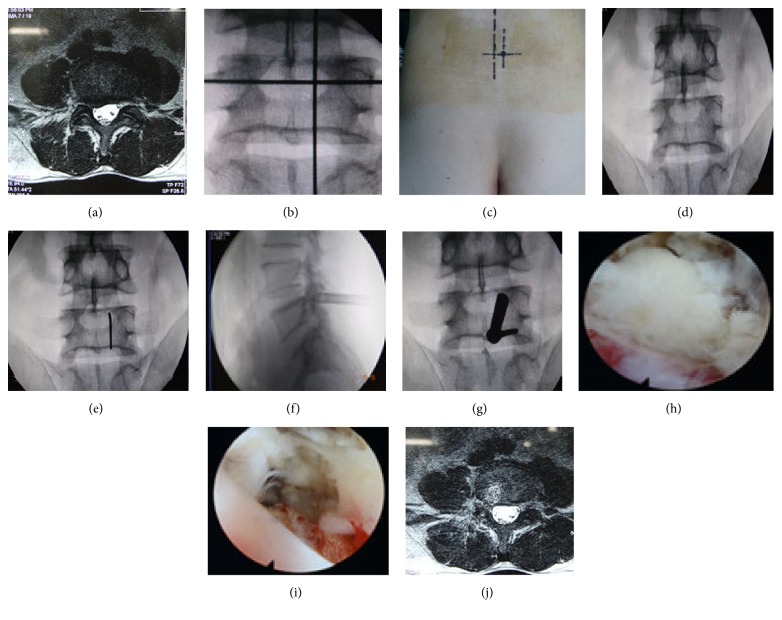
Images of patient with right-side L4/5 LDH.

All operations were performed under continuous epidural anesthesia. The patients were placed on a radiolucent orthopedic surgery bed allowing the lumbar spine to be flexed as much as possible to widen the interlaminar space. Position of the fluoroscope and the height of the operating table should be checked for convenience for the operating team. After routine sterile preparation and draping, accurate level of the incision was verified under fluoroscope, and the needle was placed with respect to the established surface piercing point (Figure 3(b)).

#### 2.3.1. Surgery of Nerve Root Shoulder (Figure 3)

The target position of puncture was located on the paracentral foraminal part of the interlaminar window and near the tip of the joints (Figure 3(d)). The needle was inserted at the piercing point stepwise to target in the spinal canal under fluoroscope guidance. A small skin incision along needle was made approximately 7 mm just enough to pass through working channel; a tapered cannulated dilator was then inserted gently along the needle to the lateral edge of the interlaminar window. The working sheath was then inserted through dilator with its beveled opening toward the spinous processes. The depth and the location of the working sheath were confirmed by fluoroscopy. We moved the dilator and placed the endoscope. Sometimes, it is difficult to perform full-endoscopic interlaminar discectomy at L4/5 or higher levels because of narrow interlaminar windows. If the anatomic osseous diameter of the interlaminar window less than 8 mm does not allow direct access into the spinal canal through the ligamentum flavum, a high-speed burr or deep laminectomy rongeur was used to resect little partial laminar bone as needed. The procedure was performed under direct visualization with normal saline irrigation at a constant rate. The ligamentum flavum was incised about 3–5 mm to enable entry into the spinal canal, so we can differentiate the vertebral canal contents. Sometimes, for ease of decompressing nerve root, it is necessary that a little bone shaving might be needed according to the position of the migrated disc. Radiofrequency (RF) probing was used to process part of the adipose tissue and blood vessels. Thereafter, by taking advantage of work channel leverage effect, we adjusted the endoscope outwards, up, or down to recognize the nerve root [4, 7–10]. The lateral recess was then enlarged by clamps. With nerve agent hook or splitting rods to probe the nerve root shoulder, the neural structures were then retracted medially and protected by rotating the beveled opening inwards 180°. We exposed the disc clearly and avoided the nerve root injury [11]. By using clamps with different angles, protrusive disc tissues were removed. Afterwards, we adjusted the endoscopic view up and down to avoid any disc residuals. The nerve root was thus fully decompressed. The ablation of nonprotrusive disc nucleus pulposus tissues and the formation of a fibrous ring due to thermosetting shrinkage were achieved with the RF cauter [8, 10]. Before end of the operation, the operation field was checked carefully to ensure that there was no dural sac damage, significant free disc organization, or active bleeding. Concurrently, good relaxation of nerve root was ensured when the nerve root could be easy mobilized from lateral to medial position. The operating system was removed, and the incised skin was sutured and covered with sterilized dressing. There was minimal blood loss and drainage was not necessary.

#### 2.3.2. Surgery of Nerve Root Axillary (Figure 4)

**Figure 4:**
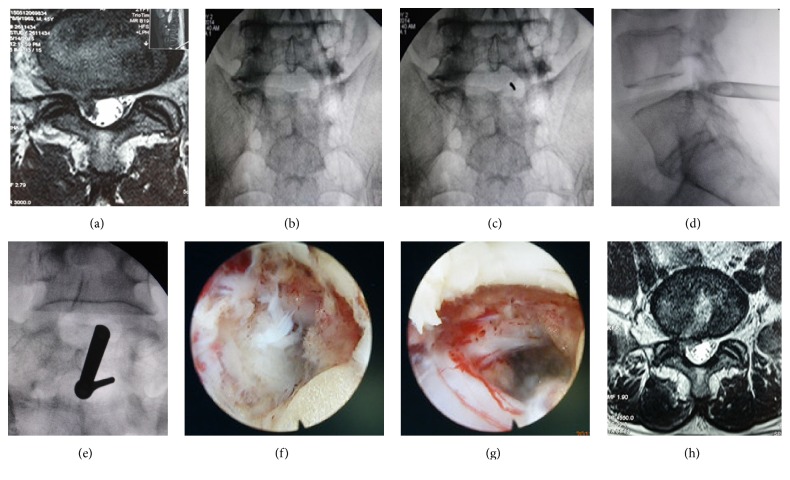
Images of patients with right-side L5S1 LDH.

The position of the vertebral plate gaps was pinpointed under C-arm fluoroscopy. The preparation of patients position was the same as previously stated. The needle was placed based on the established surface piercing point (Figure 3(b)). The target was located at the center of the interlaminar window (Figure 4(b)). After routine sterile preparation and draping, the needle was guided from the surface into the targeted points of the spinal canal, the processes of inserting dilator and a working sheath were the same as that of shoulder type stated above. After incision of the ligamentum flavum, we can expose the nerve root and its axilla. Sometimes, we can directly view the protrusive disc tissue, removing a portion of the sequestrated disc was necessary before mobilizing the nerve root, and the beveled opening of the working sheath was placed on the herniated disc with the nerve root pushed laterally or with the dura sac pushed medially. If the protrusive disc tissue is close to the center, by taking advantage of work channel leverage effect, thecal sac was pushed more medially and disclosed the protrusive disc tissue. Despite the existence of lumbar lordosis or secondary degeneration, we can adjust the endoscope to outer upper quadrant or lower outer quadrant of interlaminar bone and can recognize the nerve root, if protrusive disc tissue mildly migrated upward or downward. Bipolar electrocautery was used to obtain meticulous hemostasis and to release the soft tissue along the lateral recess. The neural structures were then retracted and protected by rotating the beveled opening inwards. The disc fragment was exposed and was resected using micropituitary instruments. The remaining procedures were the same as those for the nerve root shoulder type described in Section 2.3.1.

#### 2.3.3. Surgery of Extreme Lateral Type (Figure 5)

**Figure 5:**
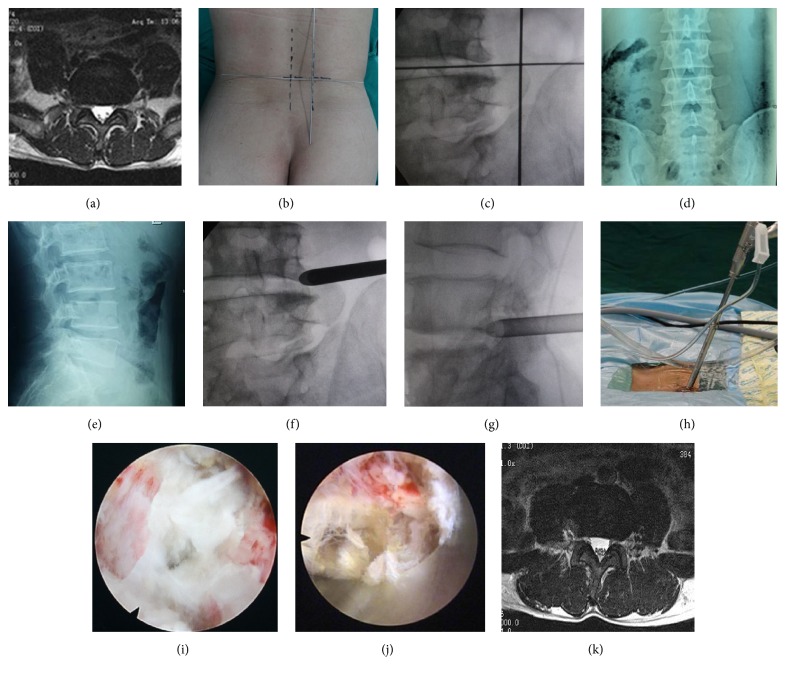
Images of patients with right-side L4/5 LDH, with symptoms in the lower and anterior side of the right thigh, knee, and medial anterior side of right leg.

Using extraforaminal puncture, the body piercing point was above and outside the transverse process on the same side (Figures 5(b) and 5(c)). The targeted sites were outer edge of inferior endplate under C-arm fluoroscopy. The side targeted point was located at posterior inferior margin of upper vertebral endplate (Figures 5(d) and 5(e)). A needle was guided from the surface piercing point along the trajectory of extraforaminal to the targets. The skin entry point was closer and the angle of needle insertion was steeper than those of transforaminal approach (Figure 5(h)). A small incision was made in the skin at the entry site, a tapered cannulated dilator was inserted gently into the extraforaminal, and a beveled opening working cannular was inserted along the dilator. Normally, the working channel is positioned in a superior-posterior position for visualization of the nerve root. And endoscope allowed direct viewing of the protrusive disc and compressed exiting nerve root. Forceps can be used to remove protrusive NP and decompressed nerve root. Afterwards, we adjusted the endoscopic view inwards, up, and down to avoid any disc residuals. The nerve root was decompressed fully. The ablation of nonprotrusive disc nucleus pulposus tissues and the formation of a fibrous ring due to thermosetting shrinkage were achieved with the RF cauter. There was minimal blood loss and drainage was not necessary. A sterile dressing was applied with one-point suture.

### 2.4. Postoperative Rehabilitation

The patients are usually given 8 to 24 hours' bed rest after operation and allowed to ambulate independently with lumbar support. They were discharged within 48 h to 96 h. Nonlaborious work was allowed after 2 weeks. Daily activities could be gradually resumed in accordance with their personal capabilities.

### 2.5. Curative Effectiveness Evaluation

A total of 60 patients received the postoperative follow-up more than 12 months by telephone interviews or outpatient review. Postoperative evaluation was conducted at 1 day, 1 month, 3 months, and final follow-up. Clinical data included perioperative parameters such as operative time, blood loss, removed disc tissue volume, and length of hospital stay. MRI was reexamined to evaluate the resection completeness of protrusive disc tissue. Visual Analogue Scale (VAS) was used to evaluate patient waist and leg pain; ODI criteria were used to evaluate the patients' daily lives. The Macnab criteria were applied to evaluate clinical curative effectiveness at 12 months.

### 2.6. Statistical Analysis

Microsoft Excel was used for data entry and formatting. SPSS 18.0 was used to study data for statistical analysis. The paired-samples* t*-test was applied to compare groups on measured data. The paired-samples *χ* ^2^ test was applied to compare groups on count data. Data are presented as mean ± standard deviation. A two-sided *P* < 0.05 was statistically significant.

## 3. Results

Forty-two were axillary type, sixteen were shoulder type, and four were extreme lateral type. Two patients were shifted to open surgery at initial time. Sixty patients completed the full-endoscopic operation successfully. The operation time was 50 min to 130 minutes (78 min on average) and there was no measurable intraoperative blood loss. The removed disc tissue volume was measured using a syringe ranged from 1.5 mL to 3.8 mL (2.4 mL on average). The time until the patient walked out of bed was 8 h to 24 h (16 h on average). The length of hospital admission after the operation was 48 h to 96 h (64 h on average). After operation, one patient of shoulder type suffered from lower limb hyperalgesia with burning sensation and five (2 cases of shoulder type and 3 cases of axillary type) had numbness of the proximal tibial side, which may be the results of thermal injury of nerve root surface by radiofrequency heat coagulation or excessive traction of nerve root. And the above symptoms acquired improvement with conservative treatment. No other blood vessel complication, bowel injury, cerebrospinal fluid leakage, or infection of intervertebral disc was discovered.

**Figure 6:**
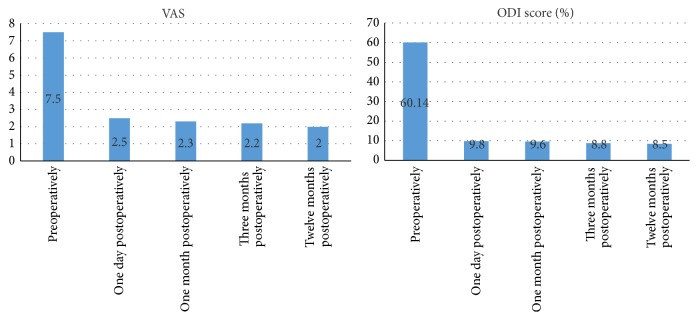
Comparison of pre- and postoperative VAS and ODI scores. Note that they decreased the most during early time of postoperative period.

These 60 patients were followed up for at least 12 months. Lumbago recurrence was observed in 2 patients 1.5 months after operation due to lumbar weight, and two patients showed leg pain during long squatting 2 months after operation. The conditions of the above 4 patients were improved with conservative treatment, and no secondary surgery was required. The preoperative VAS score was 7.5 ± 1.04 and ODI score was 60.14 ± 6.56. Twelve months after surgery, the VAS score was 2.0 ± 0.05 and ODI score was 8.5 ± 3.26 (both significant at *P* < 0.05) (Figure 6). Curative effectiveness was evaluated using the Macnab criteria after 12 months; the following results were obtained: 47 cases were excellent, 8 cases were good, 5 cases were fair, and 0 cases were poor. The excellent-to-good ratio was 91.6% (55/60) (Table 1).

## 4. Discussion

Traditional lumbar intervertebral disc excision is the standard treatment of LDH, but it entails the removal of 1/3 of the joint and the ligament flavum to fully expose the nerve tissue and protrusive disc tissues, which may inevitably lead to iatrogenic instability, epidural scar adhesion, large trauma, bleeding, long postoperative bed time, and slow recovery [4, 6]. With the assistance of full-endoscopy, the iatrogenic injury has been significantly decreased [10, 12]. The full-endoscopic technique for treatment of LDH has two accesses, transforaminal (TF) and interlaminar (IL). The full-endoscopic lumbar discectomy was first applied in the treatment of LDH using the YESS system via the posterolateral transforaminal approach [13]. Most symptomatic lumbar disc herniations, such as partial intraspinal canals and lateral disc herniations, can be successfully treated with this procedure [6, 10, 13]. However, the factors of the high-riding iliac crest (notably at L4/5 and L5S1) and the hyperplastic facet joints block the low lumbar segments. The other disadvantages include limitations in neural manipulation, the very limited foraminal working space and complicated puncture techniques, and high radiation exposure to both the surgeons and the patients. The applications of transforaminal approach are limited to removing protrusions of all intervertebral discs [6, 10]. Meanwhile, according to the anatomy study, the distance between the edge of L5 vertebral plate and L5 vertebral endplate varies from 3.0 mm to 8.5 mm; the distance between S1 vertebral plate edge and the S1 vertebral endplate is relatively constant with an average of about 13.9 mm [14]. Compared with the upper lumbar vertebral plate gap, L5S1 vertebral plate gap is very big, with an average of 31 mm (21–40 mm) [14], which makes the operation of percutaneous full-endoscopic through interlaminar discectomy very practicable. Choi et al. reported that a full-endoscopic technique through interlaminar approach could treat L5S1 disc herniation [9], and Ruetten et al. presented good effectiveness with the full-endoscopic technique through interlaminar approach for L5S1 disc herniation [10, 12]. In our study it was demonstrated that protrusive disc tissues can be safely, effectively, and adequately removed using the percutaneous full-endoscopic discectomy via targeted puncture with minimal intraoperative blood loss; there were no damage to joints and no iatrogenic instability and no infections and other complications occurrence. Excellent surgical outcomes include significant pain relief, fewer complications, minimizing soft tissue injury, and faster rehabilitation.

The applications of all types of minimally invasive technology are focused on disease tissues or other specific goals. Because the locations of the protrusive disc sites are not identical, it is difficult for single puncture approach to treat all types of protrusive disc [6, 15]. Therefore, we analyzed preoperative CT and MRI of patients, based on the main location of the herniated disc and its relationship with compressed nerve root; the site of protrusive disc was divided into shoulder type, axillary type, and extreme lateral type. We performed percutaneous full-endoscopic discectomy through center puncture of interlaminar bone, paracentral foraminal puncture of interlaminar bone, and extraforaminal puncture technique for LDH, respectively. This strategy facilitates working channel placement at the location of intervertebral disc herniation and complete disclosure of protrusive NP tissue under the endoscopic visualization. Furthermore, this acquires good nerve root decompression and achieves good effectiveness with fewer complications.

CT and MRI scan are preferred diagnostic methods for LDH. These methods can clearly show the protrusive intervertebral disc shape and location and the relationship of protrusive disc with dural sac or nerve root [15, 16]. Given patient's protrusive disc reference, different classifications were devised. With the reference from the middle line of the vertebral canal, the results can be divided into central, paracentral, and lateral types. Type of relationship with intervertebral foramen can be divided into the transforaminal and extraforaminal type. The relationship with compressed nerve root can be divided into nerve root shoulder, axillary region, and extreme lateral type [14, 16, 17]. The patients who need surgical treatment with main symptoms are caused by protrusive disc compressing corresponding nerve root. Therefore, we classified the site according to the relationship with compressed nerve root. Before operation, CT and MRI image data were carefully analyzed. We distinguished that the type of protrusive disc was either nerve root shoulder, axillary, or extreme lateral type, which provided an important reference of puncture trajectory to the position of disc herniated. Forty-two patients were axillary protrusion using center puncture of interlaminar bone, which accounted for 67.7% of this series. The location of the protrusive intervertebral disc was equivalent to the middle area of the vertebral plate gap under C-arm fluoroscopy. Therefore, the interlaminar center was a place for targeting the operation channel. The channel tip was placed into the vertebral canal and could be positioned directly at the location of the herniated intervertebral disc. This method obtained an accurate display of the protrusive disc tissue and reduced the difficulty of detecting by adjusting the channel direction, which provided a wide field of vision that was adequate for the removal of the protrusive disc tissues. Sixteen cases were of the shoulder type using paracentral foraminal puncture of interlaminar bone, accounting for 25.8% of this series. The location of the outstanding intervertebral disc was equivalent to the inner side of the lower articular process tip under C-arm fluoroscopy. Furthermore, the anatomical marks of paracentral foraminal puncture could be used as a shoulder type piercing target and direct the channel tip into the spinal canal. Subsequently, the lateral recess could be expanded with clamps. These marks could also show the protrusive intervertebral disc. Ruetten et al. applied a full-endoscopic technique through interlaminar approach for lumbar disc herniation, and the fine rate was 89.7%, with a two-year postoperative recurrence rate similar to the traditional technique [10, 12]. The postoperative recovery time was significantly reduced compared with the traditional method [17]. According to literature research of lumbar anatomy, width and height of lamina gap at L3/L4 were 13.10 ± 1.8 mm and 8.46 ± 0.65 mm, respectively, at L4/L5 were 13.80 ± 1.30 mm and 8.86 ± 0.85 mm, respectively, and at L5S1 were 15.64 ± 1.73 mm and 10.30 ± 1.2 mm, respectively [18]. Compared with L5S1 vertebral plate gap, the upper lumbar vertebral plate gap was relatively narrow. Sometimes, it is difficult to perform full-endoscopic interlaminar discectomy at L4/5 or higher levels because of narrow interlaminar windows. If the anatomic osseous diameter of the interlaminar window less than 8 mm does not allow work channel direct access into the spinal canal, a high-speed burr or deep laminectomy rongeur was used to resect a little partial laminar bone as needed. The lateral type only accounted for 4 cases, or 6.5%. We used extraforaminal puncture and acquired good results. Previous applications of Yeung's approach into the needle point were still relatively interior, and more vertical access technology was applied in the treatment of intervertebral foramen appearance in 41 patients with lumbar disc prolapse, leading to a success rate of 92% [6, 14, 19].

Sixty cases successfully completed the full-endoscopic surgery. The amount of NP removed intraoperatively had different volumes due to individual differences. The removed disc tissue volume was measured using a syringe ranged from 1.5 mL to 3.8 mL (2.4 mL on average), which completely relieved the nerve root compression. Afterwards, postoperative sciatica symptoms disappeared. No obvious recurrence was reported within one-year follow-up, and the fine rate reached 91.6%. Two patients were shifted to open surgery at initial time. The reason in one case was dural sac rupture due to excessive depth of the implanted working channel; the reason for the other case was that the protruding NP tissue could not be completely removed due to the inappropriate position of the work channel. Thus, a good working channel position is important for successful full-endoscopic surgery with proper surgical indications. Meanwhile, the percutaneous full-endoscopic targeted techniques should be specifically designed to remove the disc protrusions in various types of LDH [12, 20]. As our result demonstrated, this technique is feasible and repeatable as Ruetten proposed; targeted approach was developed to overcome lumbar disc herniation located mainly in spinal canal or extraforaminally [12, 17]. With the surgical devices and the possibility of selecting interlaminar or posterolateral to extraforaminal procedure, we can sufficiently remove the lumbar disc herniations inside and outside the spinal canal using the full-endoscopic technique. We view percutaneous full-endoscopic interlaminar or extraforaminal approach as a safe and sufficient supplementation to microsurgical procedures [21, 22]. Spine surgeons are more familiar with interlaminar approach to relieve lumbar degenerated disease. The full-endoscopic technology through interlaminar approach is easier to learn and master and reduces the steep learning curve of mastered full-endoscopic technique compared to transforaminal approach. Nonetheless, it must be remembered that difficulties can never be ruled out during the learning progress. But some preparations are necessary, such as training in an experienced spine center, and attending the cadaveric workshops could be meaningful; and “simple” cases with big interlaminar window should be operated on to begin with, in which difficulties could not be avoided thanks to manipulation technique and the anatomic situation, if problems are encountered intraoperatively and switched to a standard procedure [1, 12, 17, 21].

## 5. Conclusion

Based on the main location of the herniated disc and its relationship with compressed nerve root, we used percutaneous full-endoscopic discectomy through different puncture technique to remove the protrusive NP for LDH. Acquired benefits are fewer complications, rapid recovery, complete NP removal, effective nerve root decompression, and satisfactory cosmetic effect as well, which is a safe, effective, and rational minimally invasive spine-surgical technology with excellent clinical outcome.

## Figures and Tables

**Table 1:** Grade distribution 12-month postsurgical effect.

Indicator	Cases	Excellent	Good	Fair	Poor
Modified Macnab criteria	60	47 (78.3%)	8 (13.3%)	5 (8.5%)	0
